# The miR-17∼92a Cluster of MicroRNAs Is Required for the Fitness of Foxp3^+^ Regulatory T Cells

**DOI:** 10.1371/journal.pone.0088997

**Published:** 2014-02-11

**Authors:** Jarrod P. J. Skinner, Ashleigh A. Keown, Mark M. W. Chong

**Affiliations:** 1 St Vincent's Institute of Medical Research, Fitzroy, Victoria, Australia; 2 Department of Medicine (St Vincent's), University of Melbourne, Fitzroy, Victoria, Australia; The University of Texas Medical School at Houston, United States of America

## Abstract

By genetic inactivation of key microRNA biogenesis enzymes, we and others have previously demonstrated the critical requirement of the microRNA pathway for the differentiation and function of Foxp3^+^ regulatory T cells. In this study, we identified members of the miR-17∼92a cluster of microRNAs to be enriched in regulatory T cells. To investigate the function of this microRNA cluster, we deleted the gene specifically in Foxp3^+^ cells in mice. We found that miR-17∼92a is required for the fitness of regulatory T cells, and deficiency impacted at the level of apoptosis and proliferation of these cells. This led to a loss of Foxp3^+^ cells over time, particularly in competitive settings, and culminated in a range of immunologic perturbations. Thus, miR-17∼92a-target interactions are part of the essential microRNA networks that safeguard the regulatory T cell lineage.

## Introduction

Appropriate regulation of immune responses is critical for ensuring adequate immunity against harmful pathogens without developing uncontrolled inflammation or autoimmunity that lead to tissue damage. Regulatory T cells (Tregs) suppress the activation and proliferation of other T cells, and play a critical role in maintaining this immunologic homeostasis. Numerous T cells with suppressive activity have been described, but the best characterized are the CD4^+^CD25^+^ T cells that express the Forkhead transcription factor Foxp3. Either mutations in the gene encoding Foxp3 [1]–[3], or loss of Foxp3-expressing cells [4] results in devastating autoimmune-like lymphoproliferative disease.

Most Foxp3^+^ Tregs, at least those found in secondary lymphoid organs, arise in the thymus from a subset of thymocytes that are selected on class II major histocompatibility complexes [5]. Additionally, expression of Foxp3 can be induced in naïve CD4^+^ T cells in response to antigen stimulation in the presence of an appropriate cytokine milieu. Transforming growth factor-β is thought be a key cytokine [6], but retinoic acid may also contribute [7], [8]. Such “induced Tregs” are particularly prevalent at mucosal surfaces, such as the intestine [9].

In recent years, genome-wide approaches have provided important insights into the gene regulatory networks that enforce the Foxp3^+^ Treg lineage. Chromatin immunoprecipitation coupled to hybridization on gene arrays [10], [11], and more recently, coupled to high throughput sequencing [12], [13] have suggested that Foxp3 functions both as a transcriptional activator and repressor. The mechanisms by which this transcription factor activates or represses targets appear to be complex, involving protein complexes with other transcription factors, such as NFAT and Runx1 [14], [15], and modification of chromatin [12], [13].

Not only are transcription factors required for the regulation of gene expression networks, but also non-coding RNAs. MicroRNAs (miRNAs) are one class of non-coding RNAs that clearly have essential functions in gene regulation. These small ∼22 nt RNAs induce the translational repression and degradation of protein-coding messenger RNAs (mRNAs). The biogenesis of miRNAs requires two RNase III enzyme complexes. The nuclear complex, containing Drosha, processes long primary miRNA transcripts into stem-loop pre-miRNA intermediates [16]. The cytoplasmic complex, containing Dicer, then clips off the loop to release the mature miRNA [17]–[19]. By targeting the genes encoding Drosha or Dicer specifically in Foxp3^+^ Tregs, we and two other groups demonstrated a critical requirement of the miRNA pathway for this immune lineage [20]–[22]. Mice with Treg-specific Drosha or Dicer deficiency developed lethal lymphoproliferative disease akin to mice with mutations in the *Foxp3* gene or lacking Foxp3^+^ cells. Although Foxp3^+^ cells still developed in absence of the miRNA pathway, they were at reduced numbers. Even more striking was the complete loss of suppressive capacity by miRNA-deficient Tregs.

The studies on Drosha and Dicer deficient mice clearly established a requirement of miRNAs for the Foxp3^+^ Treg lineage. However, Tregs express many different miRNAs [23], and it was unclear which miRNAs were important. Several recent studies have since reported the function of two specific miRNAs in Tregs. miR-155 is required for inhibiting the expression of Socs1, a negative regulator of Jak-Stat signaling [24]–[26], while miR-146 inhibits the expression of Stat1 [27]. To identify other specific miRNAs that may be important for the Treg lineage, we analyzed high throughput miRNA sequencing data for miRNAs that are enriched in this lineage. Here, we report that miRNAs of the miR-17∼92a cluster are enriched in Tregs and that this cluster is important for controlling the fitness of these cells.

## Materials and Methods

### Mice and tissue preparations

Treg-specific miR-17∼92a deficient mice were generated by crossing the LoxP-flanked *Mir17∼92a^fl^* conditional allele [28] with an IRES-CreYFP allele knocked into the Foxp3 locus [29] to generate *Mir17∼92a^fl/fl^ Foxp3^CreYFP/CreYFP(Y)^* (KO) or *Mir17∼92a^fl/+^ Foxp3^CreYFP/CreYFP(Y)^* control mice. Unless otherwise stated, all animals are maintained as homozygous (females) or hemizygous (males) for the CreYFP knockin allele in order to account for any intrinsic effects of an altered *Foxp3* locus. All analyses were performed on littermate pairs, with each pair derived from a different litter. Mice carrying an IRES-GFP knocked into the Foxp3 locus have been previously described [30]. C57BL/6 and B6.SJL (marked by the CD45.1 allele) mice were purchased from Animal Resources Centre (Western Australia).

Spleen and lymph node cells were obtained by passing the organs through a 100 µm mesh. Bone marrow cells were obtained by grinding the long bones and sternum in a mortar and pestle, then filtering the cells through a 100 µm mesh. Lymphocytes were extracted from the lamina propria of colons as previously described [31].

All animal experiments were approved by the Animal Ethics Committee, Research Governance Unit of St Vincent's Hospital, and were performed under the Australian Code for the Care and Use of Animals for Scientific Purposes.

### Flow cytometry

All antibodies used for flow cytometry or cell sorting were purchased from eBioscience. Cell surface labeling of cells was performed by standard techniques. Intranuclear staining of Foxp3 was performed with the Transcription Factor Staining Buffer Set from eBioscience according the manufacturer's instructions. Annexin V staining was performed with the Annexin V Apoptosis Detection Kit from eBioscience according the manufacturer's instructions. All flow cytometry was performed on a multicolor LSRFortessa (BD Biosciences), and analyzed on Tree Star Flowjo. Statistical comparisons were performed on GraphPad Prism.

### Taqman quantitative RT-PCR

Cell sorting was performed on a FACSAria (BD Bioscience). Sorted cells were washed with cold PBS before total RNA was extracted with TRIsure reagent (Bioline) according to the manufacturer's instruction, with one modification: the RNA was precipitated with ethanol at −80°C, instead of with isopropanol on ice. The expression of specific miRNAs was quantified by Taqman MicroRNA Assay (Life Technologies) according to the manufacturer's instructions. Expression was normalized against U6 snRNA.

### Bone marrow chimeras

Total bone marrow was obtained from KO or control mice and mixed with competitor bone marrow from B6.SJL mice. B6.SJL recipient mice were irradiated twice with 6Gy separated by a 4 h interval, then injected intravenously with 2×10^6^ mixed bone marrow cells. All donor and recipient animals were at ∼8 weeks of age at the time of bone marrow transplantation. The recipient mice were allowed to recover for 8 weeks before analysis. The experimental cells were identified by CD45.2 expression, while competitor cells and any remaining recipient host cells were excluded by CD45.1 expression.

### BrdU incorporation

Mice were injected intraperitoneally with 200 µl of 10 mg/mL BrdU in PBS at 3 and 6 days prior to analysis. Cells for analysis were first stained with antibodies to cell surface markers prior to intranuclear staining for Foxp3. The cells were then refixed with the Fixation/Permeabilization buffer component of the Transcription Factor Staining Buffer Set. The cells were finally stained for BrdU incorporation using the BrdU Staining Buffer Set from eBioscience, prior to analysis.

### T cell transfers

Total CD4^+^ T cells were purified from KO and control mice by MACS separation (Miltenyi), then loaded with CFSE. 5×10^6^ cells were injected into B6.SJL recipients sublethally irradiated with 4.5Gy. Each recipient received cells from a different donor. After 8 days, the spleen and lymph nodes of each recipient mouse were harvested and cells pooled for analysis. The transferred CD4^+^Foxp3^+^ cells were identified by CD45.2 expression.

## Results

### The miR-17∼92a cluster of miRNAs are enriched in regulatory T cells

**Figure 1:**
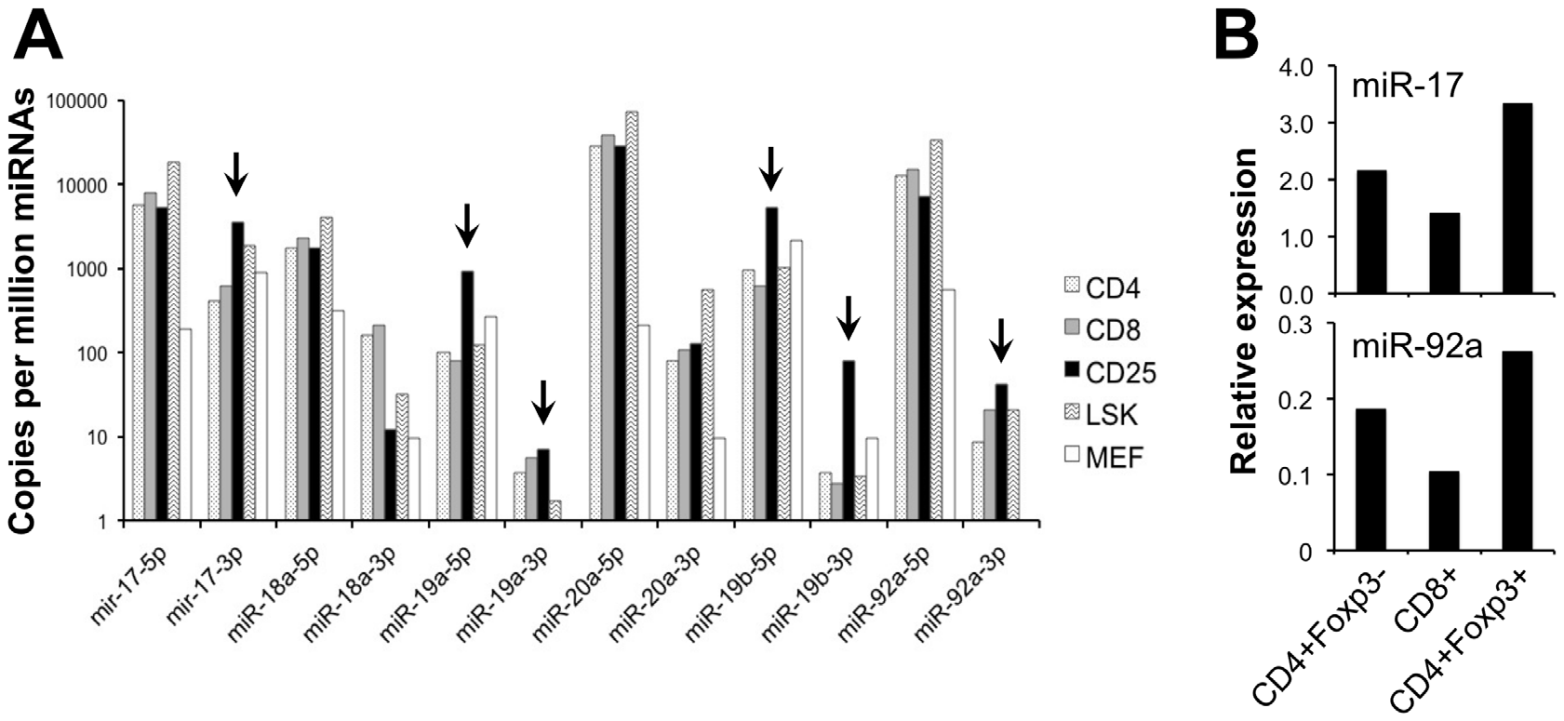
The miR-17∼92a cluster of miRNAs are enriched in Tregs. (**A**) Relative expression of the miR-17∼92a miRNAs species determined by Illumina high throughput sequencing. Shown is expression in sorted CD4^+^CD25^+^ Tregs compared to CD4^+^CD25^−^ conventional T cells, CD8^+^ T cells, bone marrow Lin^−^Sca^+^Kit^+^ stem cells, and embryonic fibroblasts (MEF) from C57BL/6 mice. The arrows indicate those species enriched in Tregs. (**B**) Quantitation of two miR-17∼92a miRNAs by Taqman-based RT-PCR. All miRNA levels were normalized to U6 snRNA. The indicated populations were sorted from *Foxp3^IRES-GFP^* mice, in which Foxp3 expression was identified by GFP fluorescence.

By targeted mutagenesis of key miRNA pathway components in mice, we and others previously demonstrated the critical requirement of miRNAs for the Foxp3^+^ Treg lineage [20]–[22]. In order to identify specific miRNAs that might be important in Tregs, we analyzed the miRNA profiles of T cell lineages that we previously generated by Illumina high throughput sequencing [23], [32]. This analysis revealed numerous miRNA species from the miR-17∼92a cluster of miRNAs to be enriched in Tregs compared to other T cell populations, bone marrow stem cells and fibroblasts (Figure 1A). The enrichment of miR-17 and miR-92a were also confirmed by Taqman-based RT-PCR assay (Figure 1B).

### The miR-17∼92a cluster is necessary for the homeostasis of regulatory T cells

**Figure 2:**
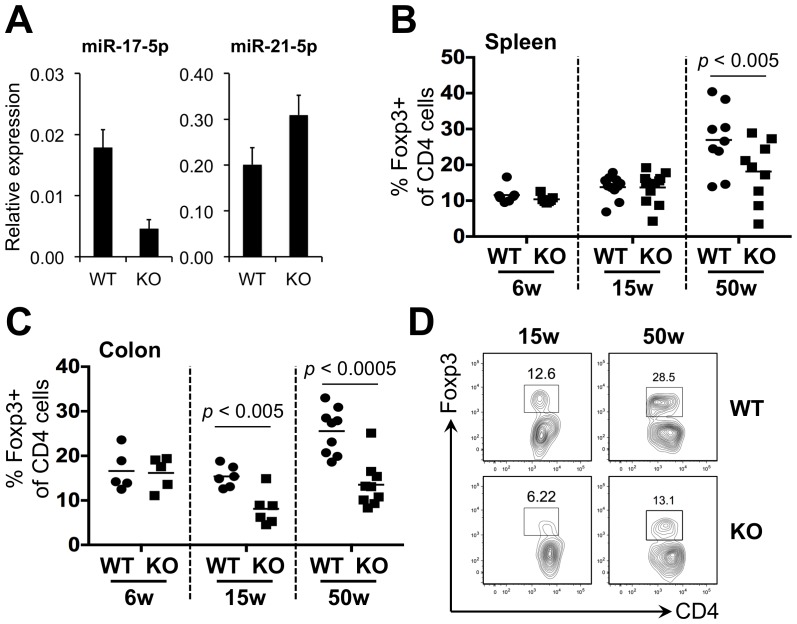
Older mice with Treg-specific miR-17∼92a deficiency display perturbations in Treg populations. (**A**) Foxp3^+^ (YFP^+^) cells were sorted from the spleens of *Mir17∼92a^fl/fl^ Foxp3^CreYFP/CreYFP(Y)^* (KO) and *Mir17∼92a^fl/+^ Foxp3^CreYFP/CreYFP(Y)^* control (WT) mice, then analyzed for miR-17 expression by Taqman-based RT-PCR. As a negative control, miR-21 expression was also measured. All miRNA levels were normalized to U6 snRNA. Frequency of Foxp3^+^ Tregs in the (**B**) spleen and (**C**) lamina propria of the colon. The data is expressed as a percentage of total CD4^+^ T cells. Littermate KO and WT pairs at ∼5 weeks (n = 5), ∼15 weeks (n = 6–8) and ∼50 weeks (n = 9) of age were analyzed. Paired *t* tests were performed for statistical comparisons. Only significant differences are indicated. (**D**) Examples of Treg profiles of mouse pairs at 15 and 50 weeks of age. Shown is the colonic lamina propria gated on TCRβ^+^ cells. Note that all TCRβ^+^ cells are CD4^+^.

The miR-17∼92 cluster of miRNAs are all derived from a single polycistronic transcript driven by a single promoter [32], [33]. To investigate the requirement of this miRNA cluster in Tregs, we generated Treg-specific miR-17∼92a deficient mice by intercrossing mice with a LoxP-flanked *Mir17∼92a^fl^* allele with a CreYFP allele expressed from the *Foxp3* locus. This resulted in a loss of miR-17∼92a cluster miRNAs but not other miRNAs (Figure 2A). In agreement with a recent report [34], Treg numbers appeared normal in the secondary lymphoid organs of young KO mice (Figure 2B). However, perturbations in Treg populations became apparent as the mice aged. Treg frequency was significantly reduced in the spleens of 50 week of KO mice. Even more dramatic was the reduction in the lamina propria of the colon. Large numbers of Tregs are normally found in the lamina propria of the colon as a result of interactions with commensal flora [9]. However, a significant reduction in Treg numbers was already evident in 15 week KO mice (Figure 2C and D).

**Figure 3:**
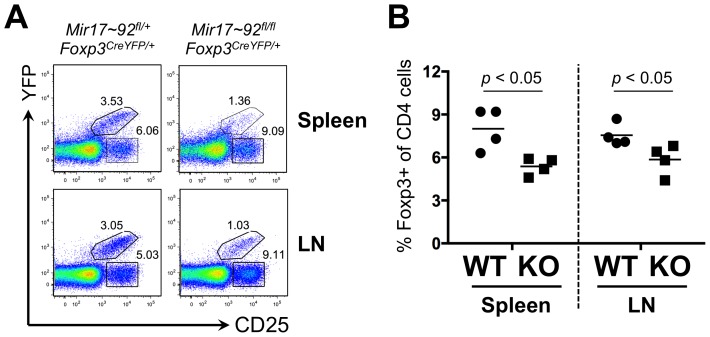
miR-17∼92a deficient Tregs have a competitive disadvantage. (**A**) Female *Foxp3^CreYFP/+^* heterozygous mice were analyzed on mir-17∼92 sufficient (fl/+) or conditional deficient (fl/fl) genetic background. Due to random X-chromosome inactivation, only half the Treg cells in these animals can potentially activate Cre expression. The Cre-expressing Tregs were identified as YFP^+^CD25^+^, whereas the non-expressing Tregs were identified as YFP^−^CD25^+^. Shown are the total CD4^+^ T cells from the spleen and lymph node of one of 4 littermate pairs analyzed. Animals were analyzed at 8 weeks of age. (**B**) Bone marrow from *Mir17∼92a^fl/fl^ Foxp3^CreYFP/CreYFP^* (KO) or *Mir17∼92a^fl/+^ Foxp3^CreYFP/CreYFP^* control (WT) mice were mixed with bone marrow from competitor CD45.1 mice, then injected into lethality irradiated CD45.1 recipient mice (n = 4). Eight weeks after reconstitution, the KO or WT cells (CD45.2^+^) in recipient mice were analyzed. Shown is the percentage of Foxp3^+^ cells within the CD4^+^ T cell compartment of the spleen and lymph node. Paired *t* tests were performed for statistical comparisons.

To explore this potential requirement of the miR-17∼92a cluster for Treg homeostasis, we investigated the fitness of Tregs in competitive settings. A clear requirement of this miRNA cluster for Treg fitness was evident in *Foxp3^CreYFP/+^* heterozygous females (Figure 3A). The *Foxp3* locus is located on the X-chromosome, and is subject to random X-inactivation to maintain gene dosage [35]. This results in only one of the two *Foxp3* alleles being transcriptionally active in any given Treg. Thus, only 50% of Tregs in a heterozygous mouse have the potential to express CreYFP. CD25^+^ Tregs that maintain the chromosome with the knockin allele can be identified as YFP^+^, whereas those that inactivate the chromosome with the knockin allele are YFP^−^. In control *Mir17∼92a^fl/+^* animals CreYFP expressing cells comprise ∼40% of the total Tregs. However, in *Mir17∼92a^fl/fl^* animals, CreYFP expressing (miR-17∼92a deficient) cells only comprise <15% of the total Tregs. That is, the miR-17∼92a deficient Tregs were outcompeted by the miR-17∼92a sufficient Tregs.

We also investigated the competitive fitness of miR-17∼92a deficient Tregs in mixed bone marrow chimeras (Figure 3B). Bone marrow from KO or control mice (marked by CD45.2) were mixed with bone marrow from B6.SJL mice (marked by CD45.1), then transplanted into lethality irradiated B6.SJL recipients. After 8 weeks, development of Tregs from KO and control bone marrow in the presence of B6.SJL competitor cells was analyzed. Consistent with the phenotype of *Foxp3^CreYFP/+^* heterozygous females, miR-17∼92a deficiency reduced the competitive fitness of Tregs in mixed bone marrow chimeras.

### Increased apoptosis and decrease proliferation of miR-17∼92a deficient Tregs

**Figure 4:**
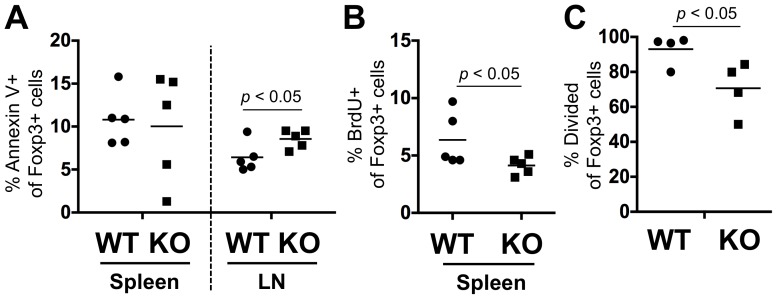
miR-17∼92a deficient Tregs display increased apoptosis and defective proliferation. (**A**) Analysis of Treg apoptosis in *Mir17∼92a^fl/fl^ Foxp3^CreYFP/CreYFP(Y)^* (KO) and *Mir17∼92a^fl/+^ Foxp3^CreYFP/CreYFP(Y)^* control (WT) mice. The Tregs from spleen and lymph node were identified as TCRβ^+^YFP^+^CD25^hi^. Littermate KO and WT pairs (n = 5) were analyzed at ∼8 weeks of age. (**B**) Analysis of Treg proliferation by BrdU incorporation. The Tregs were identified as TCRβ^+^Foxp3^+^CD25^hi^. Littermate KO and WT pairs (n = 5) were analyzed at ∼8 weeks of age. The BrdU treatment and analyses were performed over two experiments. (**C**) CFSE-labeled CD4^+^ T cells were transferred into sublethally irradiated CD45.1 recipients. After 8 days, the spleen and lymph nodes were harvested and pooled, and the transferred CD45.2 cells were analyzed for division by measuring CFSE dilution. Each data point represents a recipient transferred with cells from an individual donor KO or WT mouse (n = 4). The transfers and analyses were performed over two experiments.

We next wanted to determine if altered apoptosis or proliferation underlay the poor fitness of miR-17∼92a deficient Tregs. Apoptosis was measured by staining for Annexin V binding (Figure 4A). At stead-state, a higher frequency of Tregs was found to be Annexin V^+^ in the lymph nodes of KO mice, although it was more variable in the spleen.

We then analyzed the basal proliferation of Tregs in KO mice by measuring BrdU incorporation (Figure 4B). Mir-17∼92 deficiency resulted in a lower basal proliferation rate in Tregs.

We also analyzed the homeostatic proliferative capacity of Tregs under lymphopenic conditions (Figure 4C). Total CD4^+^ T cells were purified from KO and control mice, and labeled with CFSE. They were then adoptively transferred into sublethally irradiated B6.SJL recipients. After 8 days, cell division in the transferred cells was determined by CFSE dilution. We found that while almost all control Tregs had divided, only ∼3/4 of KO Tregs had divided during this time. Thus, we conclude that the poor fitness of miR-17∼92a deficient Tregs is most likely due to a combination of increased apoptosis and decreased proliferation of Tregs.

### Immunologic abnormalities caused by Treg-specific miR-17∼92a deficiency

**Figure 5:**
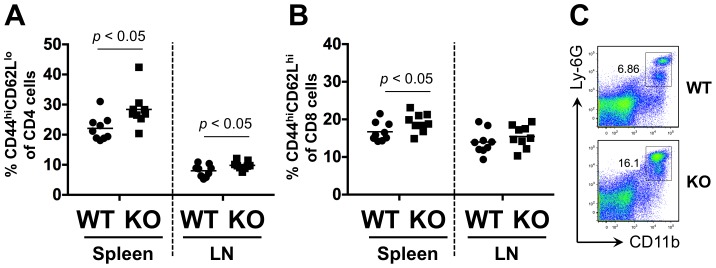
Treg-specific miR-17∼92a deficiency results in immunologic abnormalities. Littermate *Mir17∼92a^fl/fl^ Foxp3^CreYFP/CreYFP(Y)^* (KO) and *Mir17∼92a^fl/+^ Foxp3^KI/KI(Y)^* control (WT) pairs (n = 9) were analyzed at ∼15 weeks of age. T cell populations in the spleen and lymph node were stained for memory phenotype markers. Shown are (**A**) CD4^+^ T cells with an effector memory (CD44^hi^CD62L^lo^) phenotype, and (**B**) CD8^+^ T cells with an activated/central memory (CD44^hi^CD62L^hi^) phenotype. Paired *t* tests were performed for statistical comparisons. (**C**) An accumulation of large numbers of granulocytic (CD11b^+^Ly-6G^+^) cells can also be observed in the spleens of older KO mice.

Although the abnormalities caused by miR-17∼92a deficiency in Tregs were mild compared to the dramatic phenotypes caused by ablating the entire miRNA pathway, there may still be consequences of these relatively minor abnormalities. Indeed, we found that in older mice, the lack of miR-17∼92a expressing Tregs led to increases in CD4^+^ T cells with an effector memory CD44^hi^CD62L^lo^ phenotype (Figure 5A), and CD8^+^ T cells with an activated/central memory CD44^hi^CD62L^hi^ phenotype (Figure 5B). Furthermore, we noticed a substantial accumulation of CD11b^+^Ly-6G^+^ granulocytic cells in the spleen of some older mice (Figure 5C). Thus, the expression of miR-17∼92a miRNAs in Tregs is necessary for their fitness and for general immunologic homeostasis.

## Discussion

In this study, we showed that the miR-17∼92a cluster of miRNAs is enriched in Foxp3^+^ Tregs. We also showed that these miRNAs are necessary for the fitness of Tregs, regulating both proliferative capacity and apoptosis. Although it is clear that the miR-17∼92a cluster is important, the precise molecular targets of these miRNAs in Tregs remain to be determine. Multiple miRNAs are derived from this cluster, and each miRNA is predicted to recognize a large number of different targets by algorithms such as TargetScan [36]. For example, three of the Treg-enriched miRNAs, miR-17-3p, miR-19a-5p and miR-19b-5p are all predicted to target numerous signalling molecules, including multiple MAP kinases, serine/threonine kinases and phosphatases, as well as various transcription factors and cell cycle regulators. Determining which of these hundreds of potential miRNA-target interactions are important in Tregs will not be trivial. More than likely, it will be the combination of many interactions that ultimately controls the differentiation and fitness of Tregs, as well as other aspects of Treg biology. Although miR-17∼92a miRNAs are enriched in Tregs, they are still expressed at high levels in other T cell populations. Thus, many of these potential miRNA-target interactions will also important for the function of other T cells.

As mice aged, the reduced fitness of the miR-17∼92a deficient Tregs manifested as various immunologic abnormalities. These immunologic perturbations were rather subtle. In clean SPF conditions, these mice only displayed mild abnormalities in their activated/memory T cell populations. If immunologically challenged, these perturbations could predispose to inflammatory pathologies. Indeed, a previous study on the role of miR-17∼92a in IL-10-effector Tregs differentiation suggests this [34], but more work is still needed to understand the function of these miRNAs in Tregs under immunologic challenge.

A number of studies have now reported the impact of single miRNA gene deficiency in Tregs: miR-17∼92a (present study and [34]), miR-155 [24]–[26] and miR-146a [27]. Individually, loss of each of these genes resulted in clear effects on Treg biology. However, common to all is again the relatively mild phenotypes caused by these single miRNA gene mutations when compared to ablation of all miRNA biogenesis [20]–[22]. A lack of all miRNAs resulted not only in poor Treg development, but also in the production of Tregs that were completely non-functional. Dozens of miRNAs are expressed in Tregs [23], [32]. Many of these are enriched in Tregs, while others are expressed in all T cell or hematopoietic lineages. This suggests that miRNAs regulate most, if not all, critical aspects of Treg biology. The regulation of Treg fitness by the miR-17∼92a cluster is but one important function of miRNAs in Tregs. Clearly much work remains to understand the functions not only of the miR-17∼92a regulatory networks, but also of the numerous other miRNAs expressed in Tregs.

### Materials and Methods

The authors wish to thank the expert animal husbandry performed by staff of the St Vincent's BioResources Centre.

## References

[1] Bennett CL, Christie J, Ramsdell F, Brunkow ME, Ferguson PJ, et al (2001) The immune dysregulation, polyendocrinopathy, enteropathy, X-linked syndrome (IPEX) is caused by mutations of FOXP3. Nat Genet 27: 20–21. 11137993 10.1038/83713

[2] Brunkow ME, Jeffery EW, Hjerrild KA, Paeper B, Clark LB, et al (2001) Disruption of a new forkhead/winged-helix protein, scurfin, results in the fatal lymphoproliferative disorder of the scurfy mouse. Nat Genet 27: 68–73. 11138001 10.1038/83784

[3] Wildin RS, Ramsdell F, Peake J, Faravelli F, Casanova JL, et al (2001) X-linked neonatal diabetes mellitus, enteropathy and endocrinopathy syndrome is the human equivalent of mouse scurfy. Nat Genet 27: 18–20. 11137992 10.1038/83707

[4] Kim J, Lahl K, Hori S, Loddenkemper C, Chaudhry A, et al (2009) Cutting edge: depletion of Foxp3+ cells leads to induction of autoimmunity by specific ablation of regulatory T cells in genetically targeted mice. J Immunol 183: 7631–7634. 19923467 10.4049/jimmunol.0804308

[5] Fontenot JD, Rasmussen JP, Williams LM, Dooley JL, Farr AG, et al (2005) Regulatory T cell lineage specification by the forkhead transcription factor foxp3. Immunity 22: 329–341. 15780990 10.1016/j.immuni.2005.01.016

[6] Chen W, Jin W, Hardegen N, Lei KJ, Li L, et al (2003) Conversion of peripheral CD4+CD25- naive T cells to CD4+CD25+ regulatory T cells by TGF-beta induction of transcription factor Foxp3. J Exp Med 198: 1875–1886. 14676299 10.1084/jem.20030152 PMC2194145

[7] Benson MJ, Pino-Lagos K, Rosemblatt M, Noelle RJ (2007) All-trans retinoic acid mediates enhanced T reg cell growth, differentiation, and gut homing in the face of high levels of co-stimulation. J Exp Med 204: 1765–1774. 17620363 10.1084/jem.20070719 PMC2118687

[8] Mucida D, Park Y, Kim G, Turovskaya O, Scott I, et al (2007) Reciprocal TH17 and regulatory T cell differentiation mediated by retinoic acid. Science 317: 256–260. 17569825 10.1126/science.1145697

[9] Zhou L, Lopes JE, Chong MM, Ivanov, II, Min R, et al (2008) TGF-beta-induced Foxp3 inhibits T(H)17 cell differentiation by antagonizing RORgammat function. Nature 453: 236–240. 18368049 10.1038/nature06878 PMC2597437

[10] Zheng Y, Josefowicz SZ, Kas A, Chu TT, Gavin MA, et al (2007) Genome-wide analysis of Foxp3 target genes in developing and mature regulatory T cells. Nature 445: 936–940. 17237761 10.1038/nature05563

[11] Marson A, Kretschmer K, Frampton GM, Jacobsen ES, Polansky JK, et al (2007) Foxp3 occupancy and regulation of key target genes during T-cell stimulation. Nature 445: 931–935. 17237765 10.1038/nature05478 PMC3008159

[12] Samstein RM, Arvey A, Josefowicz SZ, Peng X, Reynolds A, et al (2012) Foxp3 exploits a pre-existent enhancer landscape for regulatory T cell lineage specification. Cell 151: 153–166. 23021222 10.1016/j.cell.2012.06.053 PMC3493256

[13] Katoh H, Qin ZS, Liu R, Wang L, Li W, et al (2011) FOXP3 orchestrates H4K16 acetylation and H3K4 trimethylation for activation of multiple genes by recruiting MOF and causing displacement of PLU-1. Mol Cell 44: 770–784. 22152480 10.1016/j.molcel.2011.10.012 PMC3243051

[14] Wu Y, Borde M, Heissmeyer V, Feuerer M, Lapan AD, et al (2006) FOXP3 controls regulatory T cell function through cooperation with NFAT. Cell 126: 375–387. 16873067 10.1016/j.cell.2006.05.042

[15] Ono M, Yaguchi H, Ohkura N, Kitabayashi I, Nagamura Y, et al (2007) Foxp3 controls regulatory T-cell function by interacting with AML1/Runx1. Nature 446: 685–689. 17377532 10.1038/nature05673

[16] Lee Y, Ahn C, Han J, Choi H, Kim J, et al (2003) The nuclear RNase III Drosha initiates microRNA processing. Nature 425: 415–419. 14508493 10.1038/nature01957

[17] Hutvagner G, McLachlan J, Pasquinelli AE, Balint E, Tuschl T, et al (2001) A cellular function for the RNA-interference enzyme Dicer in the maturation of the let-7 small temporal RNA. Science 293: 834–838. 11452083 10.1126/science.1062961

[18] Ketting RF, Fischer SE, Bernstein E, Sijen T, Hannon GJ, et al (2001) Dicer functions in RNA interference and in synthesis of small RNA involved in developmental timing in C. elegans. Genes Dev 15: 2654–2659. 11641272 10.1101/gad.927801 PMC312808

[19] Grishok A, Pasquinelli AE, Conte D, Li N, Parrish S, et al (2001) Genes and mechanisms related to RNA interference regulate expression of the small temporal RNAs that control C. elegans developmental timing. Cell 106: 23–34. 11461699 10.1016/s0092-8674(01)00431-7

[20] Chong MM, Rasmussen JP, Rudensky AY, Littman DR (2008) The RNAseIII enzyme Drosha is critical in T cells for preventing lethal inflammatory disease. J Exp Med 205: 2005–2017. 18725527 10.1084/jem.20081219 PMC2526196

[21] Liston A, Lu LF, O'Carroll D, Tarakhovsky A, Rudensky AY (2008) Dicer-dependent microRNA pathway safeguards regulatory T cell function. J Exp Med 205: 1993–2004. 18725526 10.1084/jem.20081062 PMC2526195

[22] Zhou X, Jeker LT, Fife BT, Zhu S, Anderson MS, et al (2008) Selective miRNA disruption in T reg cells leads to uncontrolled autoimmunity. J Exp Med 205: 1983–1991. 18725525 10.1084/jem.20080707 PMC2526194

[23] Chong MM, Zhang G, Cheloufi S, Neubert TA, Hannon GJ, et al (2010) Canonical and alternate functions of the microRNA biogenesis machinery. Genes Dev 24: 1951–1960. 20713509 10.1101/gad.1953310 PMC2932976

[24] Lu LF, Thai TH, Calado DP, Chaudhry A, Kubo M, et al (2009) Foxp3-dependent microRNA155 confers competitive fitness to regulatory T cells by targeting SOCS1 protein. Immunity 30: 80–91. 19144316 10.1016/j.immuni.2008.11.010 PMC2654249

[25] Yao R, Ma YL, Liang W, Li HH, Ma ZJ, et al (2012) MicroRNA-155 modulates Treg and Th17 cells differentiation and Th17 cell function by targeting SOCS1. PLoS One 7: e46082. 23091595 10.1371/journal.pone.0046082 PMC3473054

[26] Kohlhaas S, Garden OA, Scudamore C, Turner M, Okkenhaug K, et al (2009) Cutting edge: the Foxp3 target miR-155 contributes to the development of regulatory T cells. J Immunol 182: 2578–2582. 19234151 10.4049/jimmunol.0803162

[27] Lu LF, Boldin MP, Chaudhry A, Lin LL, Taganov KD, et al (2010) Function of miR-146a in controlling Treg cell-mediated regulation of Th1 responses. Cell 142: 914–929. 20850013 10.1016/j.cell.2010.08.012 PMC3049116

[28] Ventura A, Young AG, Winslow MM, Lintault L, Meissner A, et al (2008) Targeted deletion reveals essential and overlapping functions of the miR-17 through 92 family of miRNA clusters. Cell 132: 875–886. 18329372 10.1016/j.cell.2008.02.019 PMC2323338

[29] Rubtsov YP, Rasmussen JP, Chi EY, Fontenot J, Castelli L, et al (2008) Regulatory T cell-derived interleukin-10 limits inflammation at environmental interfaces. Immunity 28: 546–558. 18387831 10.1016/j.immuni.2008.02.017

[30] Bettelli E, Carrier Y, Gao W, Korn T, Strom TB, et al (2006) Reciprocal developmental pathways for the generation of pathogenic effector TH17 and regulatory T cells. Nature 441: 235–238. 16648838 10.1038/nature04753

[31] Chong MM, Simpson N, Ciofani M, Chen G, Collins A, et al (2010) Epigenetic propagation of CD4 expression is established by the Cd4 proximal enhancer in helper T cells. Genes Dev 24: 659–669. 20360383 10.1101/gad.1901610 PMC2849123

[32] Kirigin FF, Lindstedt K, Sellars M, Ciofani M, Low SL, et al (2012) Dynamic microRNA gene transcription and processing during T cell development. J Immunol 188: 3257–3267. 22379031 10.4049/jimmunol.1103175 PMC3934760

[33] He L, Thomson JM, Hemann MT, Hernando-Monge E, Mu D, et al (2005) A microRNA polycistron as a potential human oncogene. Nature 435: 828–833. 15944707 10.1038/nature03552 PMC4599349

[34] de Kouchkovsky D, Esensten JH, Rosenthal WL, Morar MM, Bluestone JA, et al (2013) microRNA-17-92 regulates IL-10 production by regulatory T cells and control of experimental autoimmune encephalomyelitis. J Immunol 191: 1594–1605. 23858035 10.4049/jimmunol.1203567 PMC4160833

[35] Dupont C, Gribnau J (2013) Different flavors of X-chromosome inactivation in mammals. Curr Opin Cell Biol 25: 314–321. 23578369 10.1016/j.ceb.2013.03.001

[36] Lewis BP, Burge CB, Bartel DP (2005) Conserved seed pairing, often flanked by adenosines, indicates that thousands of human genes are microRNA targets. Cell 120: 15–20. 15652477 10.1016/j.cell.2004.12.035

